# Real-World Use of Carvedilol in Children With Dilated Cardiomyopathy: Long-Term Effect on Survival and Ventricular Function

**DOI:** 10.3389/fped.2022.845406

**Published:** 2022-04-01

**Authors:** Rachele Adorisio, Nicoletta Cantarutti, Marco Ciabattini, Antonio Amodeo, Fabrizio Drago

**Affiliations:** ^1^Advanced Cardiovascular Therapies, Department of Cardiology, Cardiac Surgery, Heart and Lung Transplant, Bambino Gesù Children Hospital and Research Institute, Rome, Italy; ^2^Pediatric Cardiology and Arrhythmias/Syncope Units, Department of Cardiology, Cardiac Surgery, Heart and Lung Transplant, Bambino Gesù Children Hospital and Research Institute, Rome, Italy; ^3^Clinical Trial Center, University Department of Pediatrics, Bambino Gesù Children Hospital and Research Institute, Rome, Italy

**Keywords:** β-blockers, carvedilol, dilated cardiomyopathy, children, heart failure

## Abstract

**Background:**

Carvedilol is recommended for chronic heart failure (HF) treatment in children. However, the ideal dosage and administration are not standardized, and data on its long-term effects are lacking. This study aimed to assess the effect of a high dosage regimen of carvedilol on cardiac outcomes in children with HF.

**Methods:**

We conducted a retrospective cohort study including all children with HF and dilated cardiomyopathy. We analyzed medical records before starting treatment, at 1 and 3 years after reaching the maximum therapeutic dosage. All data were compared with a historical control group. Kaplan–Meier analysis and Cox proportional hazard regression have been used to evaluate the effect of high dosage carvedilol therapy. The main outcome was a composite of all-cause mortality and heart transplant.

**Results:**

One hundred thirty-five were included in the study and 65 treated with a high dosage of carvedilol regimen (up to 1 mg/kg/day). Heart rate reduction (mean reduction 30%, *p* < 0.0001) and ejection fraction improvement (32 ± 9.4 vs. 45. ± 10.1%, *p* < 0.0001) were statistically significant in those. Long-term survival and freedom from heart transplant were significantly improved in those treated with high dosage carvedilol therapy (*p* = 0.00001).

**Conclusions:**

Treatment with the high dosage of carvedilol, in addition to standard HF therapy, significantly improves ventricular function and survival in children with dilated cardiomyopathy and chronic HF.

## Introduction

Chronic heart failure (HF) due to dilated cardiomyopathy (DCM) remains a significant medical challenge in a pediatric population, leading to transplantation or death in 40% of children and adolescents (1). There is a paucity of evidence about the pharmacological management of this population, and few prospective studies have been conducted so far. Based on adult experience (2–4), a consensus of experts suggests using β-blockers and angiotensin-converting enzyme (ACE) inhibitors to treat children with HF (5). The first trial designed to assess the efficacy of carvedilol in pediatric HF failed to demonstrate its objective (6). In common clinical practice, this drug is used at different dosages and ways of administration (7). Pharmacokinetic (PK) studies (8, 9) showed that a higher dose is needed in children to reach the same efficacy as in the adult population, suggesting that the efficacy of treatment may be related to dose exposure.

The present study aimed to evaluate the efficacy of different dosages of carvedilol in children and adolescents with symptomatic chronic HF due to DCM at long-term follow-up.

## Methods

### Study Design

A retrospective cohort study has been performed to evaluate the effect of carvedilol at high dosage in HF children affected by DCM at long-term follow-up. All children diagnosed with DCM referred to Bambino Gesù Hospital from 2013 to 2017 were assessed for inclusion in the cohort.

Among those, patients matching the following criteria have been included: (1) age at clinical presentation ≥1 year and <18 years; (2) diagnosis of DCM, according to the current cardiomyopathy classification (10); (3) left ventricular (LV) ejection fraction (EF) < 45%; (4) chronic HF treatment with an ACE inhibitor started at least 72 h before; (5) period of follow-up > 24 months.

All cases of acute lymphocytic myocarditis and/or inflammatory cardiomyopathy assessed by endomyocardial biopsy and/or cardiac magnetic resonance imaging were excluded from the study.

The medical records of the included patients were assessed through a retrospective review of clinical charts.

The individual patient follow-up started from the first medical record available of the Bambino Gesù Children Hospital in which an LVEF < 45% was recorded and lasted to the date of death/heart transplant or to the last available medical record within the study follow-up.

The following data have been collected at 1 and 3 years of follow-up: New York Heart Association/Ross functional class, blood pressure measurement, standard electrocardiogram, echocardiogram, brain natriuretic peptide (BNP) dosage, and pharmacological treatment.

Two blinded expert cardiologists separately reanalyzed standard electrocardiograms and echocardiograms.

Echocardiographic images in two dimensions were digitally stored, and measurements were made offline. LV volumes (end-diastolic, LVEDV, end-systolic, LVESV) were analyzed on three consecutive beats; apical four-chamber and two-chamber views were used in accordance with guidelines of the American Society of Echocardiography (11). Wall thickness and chamber dimensions were evaluated from the two-dimensional parasternal long-axis view or M-mode short-axis view at the mid-ventricular level on three consecutive beats (12). LVEF was calculated using the biplane Simpson formula (11).

Clinical outcomes (death and heart transplant) were collected throughout the follow-up.

### Carvedilol Exposure

Patients matching the inclusion criteria mentioned earlier and treated with carvedilol were considered in this group. Carvedilol was uptitrated every 2 weeks to reach a high dosage (≥ 0.8 ≤ 1 mg/kg/day). Reason for interruption of uptitration was noticed and way of administration and uptitration assessed. All concomitant drugs (i.e., ACE inhibitors, diuretics, and digoxin) have been recorded. All adverse effects reported were noted.

### Control Group

To evaluate the effect of carvedilol, we compared patients exposed to carvedilol regimen with patients undergoing other pharmacological treatment only (i.e., diuretics, digoxin, and ACE inhibitors). We considered as “control group” all patients who did not undergo carvedilol therapy or other types of β-blockers. This group of patients was extrapolated by our historical database, followed by primary cardiologists between 2003 and 2013. Clinical characteristics were comparable and reported in Table 1.

**Table 1:** Characteristics of groups.

	**Carvedilol** **group = 65**	**Control** **group = 70**	***p*-value**
Age, yrs (mean)	8.7 ± 8.9	5.2 ± 4.8	0.25
Sex (M)	38	49	0.51
Ross/NYHA ≥ II	46	50	0.97
BNP pre	91.6 ±128.9	102 ± 110.2	0.89
BNP post	45.2 ± 74.3	330 ± 143.9	**<0.001**
HR pre	107.8 ± 29.4	110 ± 12.3	0.79
HR post	72.4 ± 12.9	120 ± 2.4	**0.05**
LVEF (%) pre	32 ± 9.4	30 ± 6.9	0.09
LVEF (%) post	45 ± 10.1	24.5 ± 18.5	**<0.001**
**Pharmacological treatment**			
Beta-blockers (Carvedilol)	65	0	**<0.001**
ACE-inhibitors	65	70	1
Diuretics	40	49	0.63
Aldosterone inhibitors	38	43	0.86
Anti-platelets	33	49	0.26

*BNP, brain natriuretic peptide; HR, heart rate; LVEF, left ventricular ejection fraction. Bold values indicate that they are statistically significant*.

### Efficacy Endpoints

The primary outcome measure of the study was a composite of all-cause mortality and heart transplantation.

Secondary endpoints were echocardiographic measures (LVEF, LVEDV, and LVESV) and clinical measurements (heart rate and BNP values).

### Statistical Analysis

Continuous variables are presented as mean values and standard deviations. Categorical variables are expressed as absolute numbers and percentages. Paired t-test was used to compare continuous variables. Univariate and multivariate analyses were performed by Cox regression analysis. All variables that resulted significantly in univariate analysis were entered in multivariate analysis to determine the independent predictors of long-term outcomes. Results are expressed as the hazard ratio, with 95% confidence intervals (CIs). Survival data and freedom from adverse events (death and heart transplant) were analyzed and graphically reported by the Kaplan–Meier method. Patients who did not experience any events were censored at the time of the last follow-up. The difference in survival between groups was analyzed using the log-rank test. A *p* < 0.05 was considered significant. All statistical analyses were performed by SPSS Statistics 21 (IBM Corporation, Armonk, NY, USA).

## Results

One hundred thirty-five matched the inclusion criteria and were included in our cohort. The mean (standard deviation) duration of follow-up was 4.8 (±1.4) years. According to carvedilol exposure, we divided all cases into two groups: patients on carvedilol (*n* = 65) and patients not treated with carvedilol “control group” (*n* = 70). In the carvedilol group, a high dosage of carvedilol was achieved in 60 (92.3%).

The groups did not differ significantly at baseline in clinical and imaging characteristics (Table 1). No differences were encountered for concomitant treatment between the two groups, except for carvedilol exposure. The reason for uptitration interruption in the carvedilol group was LVEF improvement (15.2%).

### Clinical Outcome

No cases of hypotension, bradycardia, atrioventricular block, bronchoreactivity, or hypoglycemia have been observed in the carvedilol group.

No adverse outcomes (death or cardiac transplantation) occurred in any patients on treatment with high dosage carvedilol. All patients in this group survived, and four of four patients were delisted from the transplant waiting list. In the carvedilol group, all patients changed New York Heart Association/Ross class except one (1.5%), who remained in functional class III; among the remaining 64 patients, the functional class decreased to class II in 37% and to class I in 61.5%. No changes in drug regimen over time were reported.

**Figure 1:**
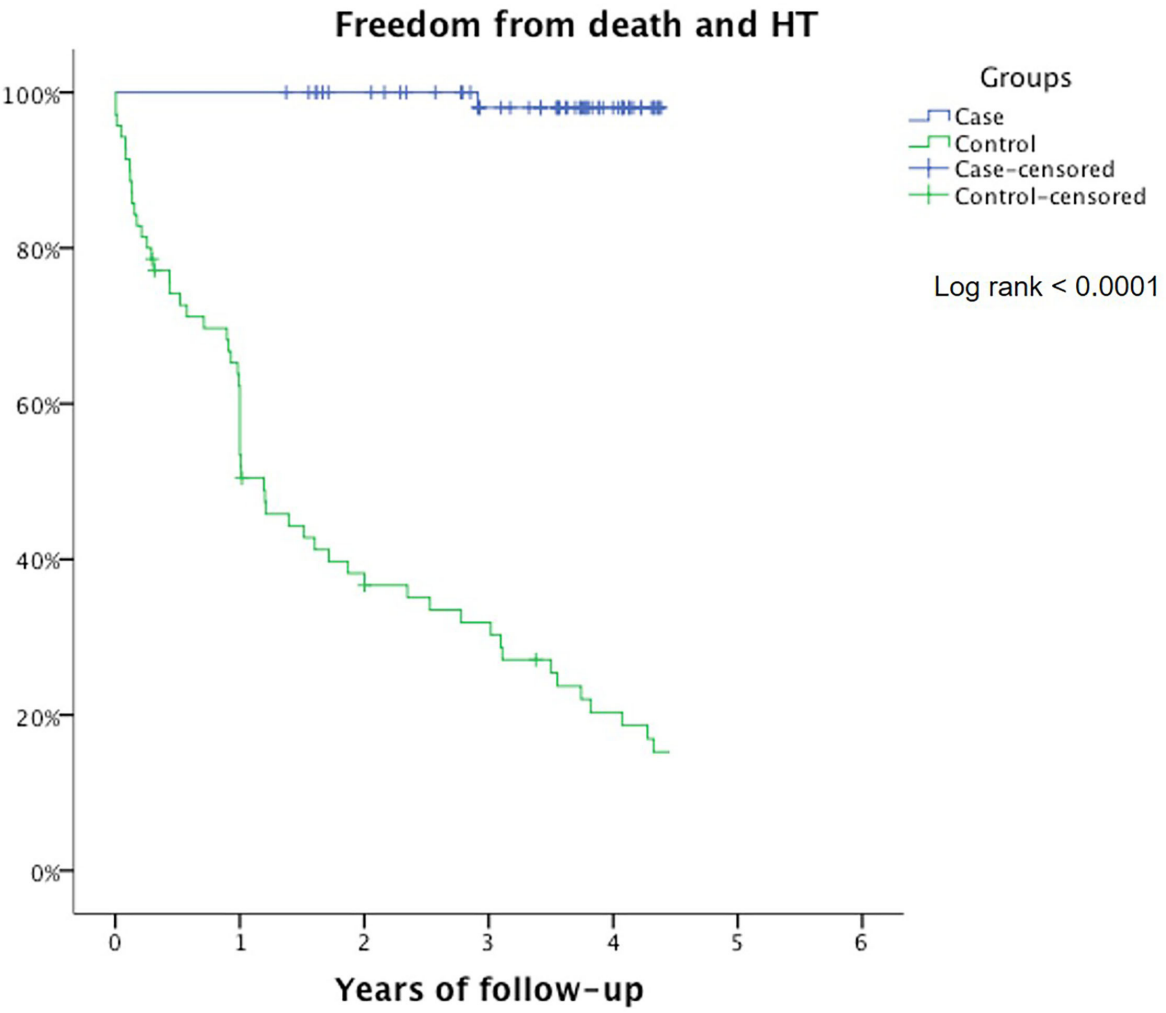
Freedom from death/heart transplant represented by Kaplan–Meier curves in carvedilol group (blue) and control group (green).

In the control group, 5 patients died (7%), and 24 patients were transplanted (34%) after 1 year of follow-up. The multivariate Cox regression analysis showed that the use of β-blockers and reduction in heart rate are the only independent predictor of long-term survival and freedom from heart transplant (*p* < 0.0001) (Table 2). Kaplan–Meier curve shows that survival is 100% for the carvedilol group after 1 year and 98% after 3 years of follow-up, compared with 28% in the control group (log-rank <0.0001) (Figure 1).

**Table 2:** Results of univariable and multivariable Cox regression for long-term outcomes.

**Variable**	**Univariable**			**Multivariable Cox**		
	**HzR**	**95% CI**	***P* value**	**HzR**	**95% CI**	***P* value**
Sex (M)	0.730	0.447–1,192	0.208			
Age	0.958	0.920–0.998	**0.039**	0.996	0.946–1,049	0.884
Ross/NYHA I (ref)			0.764			
Ross/NYHAII	1.030	0.709–1,497	0.876			
Ross/NYHA III	0.888	0.638–1,236	0.480			
HR pre	1.006	0.998–1,015	0.126			
HR post	1.034	1,025–1,044	**0.000**	1.007	0.994–1,020	0.266
LVEF pre	0.983	0.954–1,013	0.273			
Beta-blockers	0.011	0.002–0.080	**0.000**	0.016	0.002–0.129	**0.000**
ACE-inhibitors						
Diuretics	1.383	0.777–2,463	0.270			
Aldosterone inhibitors	1.350	0.796–2,292	0.266			
Anti-platelets	1.884	1,057–3,359	**0.032**	1.047	0.584–1,878	0.876

*HR, heart rate; LVEF, left ventricular ejection Fraction; HzR, hazard ratio; 95% CI, 95% confidence interval. Bold values indicate that they are statistically significant*.

A significant difference between the high dosage of carvedilol and the control group (*p* < 0.0001) was reported.

### Heart Rate and Cardiac Imaging

No significant differences between mean HR and EF at baseline were observed between the two groups (Table 1).

In the carvedilol group, the mean level of serum BNP at 1-year follow-up decreased from the baseline value of 91.6 to 45 pg/ml; BNP level in the control group at the same time increased from a mean baseline value of 102 to 330 pg/ml (*p* < 0.001).

**Figure 2:**
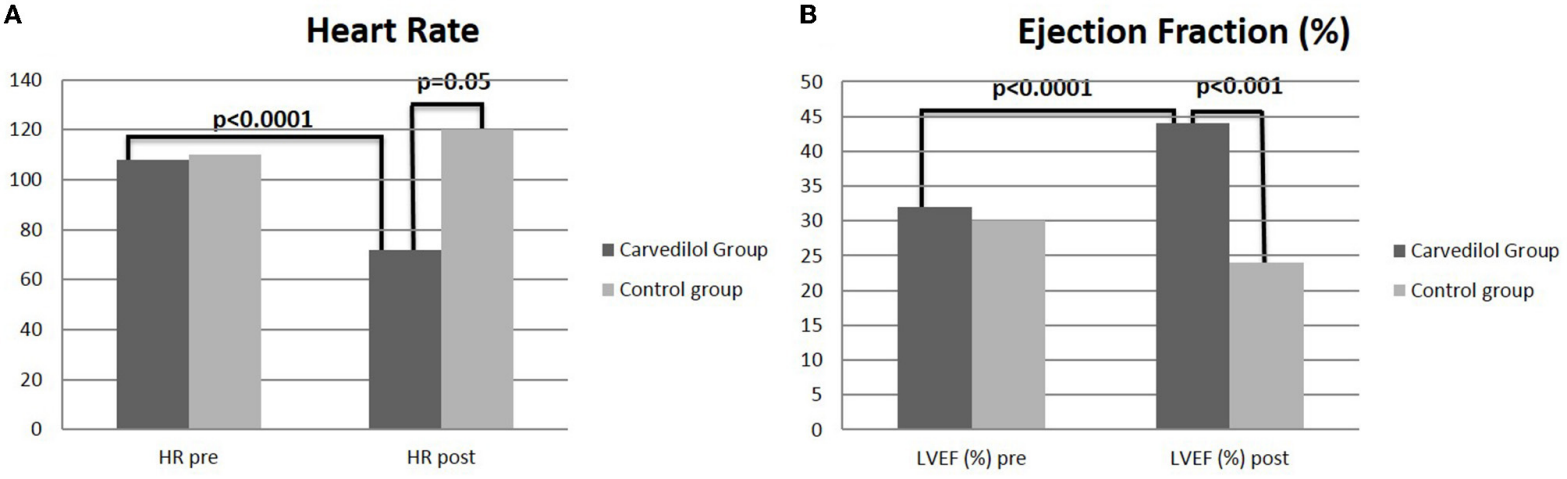
T-test analysis between carvedilol and control group on heart rate **(A)** and left ventricular ejection fraction **(B)** at baseline and at 1 year of follow-up.

The mean HR reduction calculated at the achievement of the maximum dose of carvedilol was 30%, with a mean of 72.3 bpm (*p* < 0.0001) (Table 1). In the control group, no differences in HR from baseline to the end of FU were observed (Figure 2).

**Figure 3:**
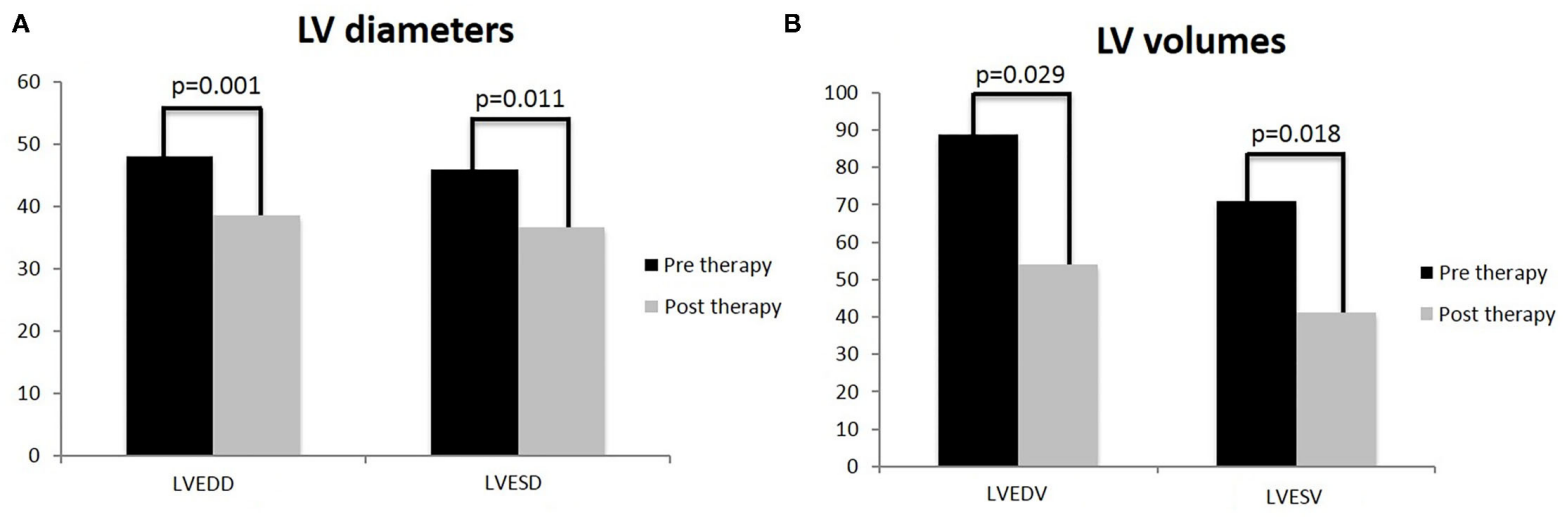
T-test analysis on left ventricle diameters and volumes in children treated with carvedilol at baseline and at achievement of maximum dose. Left ventricular end-diastolic diameter (LVEDD) and left ventricular end-systolic diameter (LVESD) **(A)**; left ventricle volumes: left ventricular end-diastolic volume (LVEDV) and left ventricular end-systolic volume (LVESV) **(B)**. Values are represented by mean.

In the carvedilol group, mean LVEDV and LVESV and LV diameters (end-diastolic, LVEDD, end-systolic, LVESD) at baseline and at the achievement of the maximum dose differed significantly (Figure 3).

**Figure 4:**
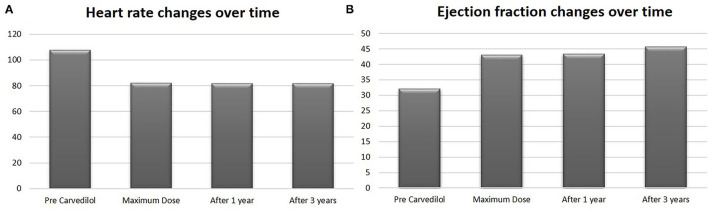
Heart rate and ejection fraction values in carvedilol group at baseline, at maximum dose, after 1 year and after 3 years of follow-up. Values are represented by mean.

In the carvedilol group, the mean EF at the maximum dose achievement was 43.9% (*p* < 0.0001) (Figure 4), with a mean improvement of LV function of 11.9% compared with baseline.

After 3 years of follow-up, the mean EF was 45.6% (Figure 4) in the carvedilol group, whereas that in the control group was 24.5% (*p* < 0.001) (Figure 2).

### Subgroup Analysis in the Carvedilol Group

According to age and type of DCM, we observed several differences among the carvedilol group. Patients younger than 6 years appeared to have the most pronounced increase in mean LVEF (20.6%) when treated with the high dosage of carvedilol. Accordingly, LVEDD and LVESV reductions from baseline were statistically significant only in patients younger than 6 years (*p* = 0.038 and *p* = 0.007, respectively), whereas LVEDV showed a trend toward significance (*p* = 0.06). In the group > 12 years, we do not observe the same significant difference. This is due to seven Duchenne patients whose ventricular function remained unchanged or improved <10%. The idiopathic DCM group showed a significant reduction of LVEDD (*p* = 0.017, CI 0.5–4.3), but LVEDV and LVESV did not decrease significantly despite showing a positive trend (*p* = 0.093, CI −4 to 46 and *p* = 0.073, CI −2 to 43). A significant improvement of LVEF was recorded in all groups, regardless of the DCM etiology.

## Discussion

This study evaluated the efficacy of a high dosage carvedilol regimen in long-term follow-up in children with chronic HF due to DCM.

Our analysis shows that a high dosage of carvedilol (1 mg/kg/day) is effective in reducing major cardiovascular events, such as death or orthotopic heart transplantation, when compared with only ACE inhibitors and diuretics. Multivariate Cox regression demonstrates that carvedilol is the only independent protective factor for improving prognosis. This finding is well displayed by Kaplan–Meier curves and evidenced by log-rank analysis, confirming how survival is significantly better in the treated group, especially in the long-term follow-up. The efficacy seems to be confirmed in the clinical scenario, as all four patients on the waiting list for transplant have been delisted. Ventricular function resulted also significantly improved during follow-up in those treated with intermediate and high dosage. More important, this beneficial effect of carvedilol with these dosages appears to be sustained over the years. Considering a 3-year period, these data confirm that a high dosage of carvedilol may have a significant impact on ventricular function recovery.

So far, different studies have been published on carvedilol use in pediatric HF, but to our knowledge, no one exceeded 6 months of follow-up. In the clinical arena, carvedilol dosage in pediatric patients ranged between 0.2 and 0.8 mg/kg/day (11, 13–18). With these dosages, different studies reported significant improvement in EF, shortening fraction and clinical status within a 6-month period (11, 13–18). Nevertheless, the first published prospective, multicenter, randomized, double-blind, controlled pediatric clinical trial (6) on carvedilol failed to support a beneficial effect of this β-blocker. This study included two carvedilol arms with different dosages of 0.4 and 0.8 mg/kg/day. This trial did not show a significant improvement in cardiovascular outcomes of children and adolescents with symptomatic systolic HF, although there was a trend in significance for DCM patients who took a higher dosage. Notably, in this study: (1) both patients with DCM or congenital heart disease were enrolled; (2) HR showed a significant decrease from baseline of around 10%; (3) plasma concentrations of carvedilol resulted lower than that derived in adults. The lack of carvedilol efficacy reported by Shaddy et al. could be then determined by a heterogeneous population and drug exposure levels below the efficacy threshold.

More recently, Huang et al. (11) performed a randomized controlled trial on carvedilol, including only pediatric patients with DCM. They described that the major clinical improvement was in patients who reached the highest dose of 0.8 mg/kg/day. In this study, beneficial effect on ventricular function and relief of symptoms was registered after 6 months of therapy, depending on the dose reached. Interestingly, the reduction of heart rate was a criterion for discontinuing uptitration.

Our data show that a high dosage of carvedilol reduces heart rate significantly as an induced negative chronotropic effect. Uptitration of the drug reaching 1 mg/kg/day, as required by reduction of heart rate and/or improvement of ventricular function, showed to have a beneficial effect. In the adult population, HR is considered an important key point for treatment strategy. According to literature, a meta-regression analysis demonstrated that the beneficial effect of β-blockers on mortality was achieved when HR was reduced to 60 bpm in an adult with HF (19), corresponding to a 20–25% HR reduction from baseline. In the pediatric population, recent studies (20, 21) on the use of ivabradine suggested that a reduction of HR could be beneficial. Our data are consistent with these pieces of evidence, as the negative chronotropic effect of a high carvedilol dosage regimen was reported in all patients with improvement of ventricular function regardless of different ages and DCM etiologies. Among all β-blockers, carvedilol is able to inhibit both β_2_- and α_1_-adrenergic receptors. Such actions are noteworthy, as children are more sensitive to potassium depletion for the concomitant use of diuretics. This consideration might be especially important for children with HF, whose potassium balance may be disturbed by furosemide (22, 23).

The importance of prescribing a high dosage of carvedilol is also supported by PK studies (8). Albers et al. showed that the values of PK measures in pediatric patients depend on age and weight. Dose simulations revealed that younger patients need to be treated with higher doses to reach the same drug exposure as adults. Albers et al. (8) also demonstrated that children required higher dose exposure to obtain the same plasma concentration as adults with HF. On the basis of these efficacy and PK pieces of evidence (8, 22), this study was designed to evaluate how to find the target dose. We did obtain the achievement of 1 mg/kg/day in most of our patients through a slow uptitration protocol and heart rate <60 bpm, as drug dosage increased stop criteria. We did not observe any adverse effects, such as symptomatic hypotension and/or bradycardia.

Our subgroup analysis by age also confirmed the evidence obtained from PK simulations (8, 9). The subgroup analysis by etiology showed the major improvements of EF in idiopathic and sarcomeric DCM. Our data show that high dosage carvedilol might be effective in improving survival and ventricular function, especially in younger patients.

## Limitations of the Study

This study was limited by the fact that data were evaluated retrospectively. In our analysis, we excluded subjects with age <1 year. This group of patients has a poor prognosis. Applying a definition of chronic HF is difficult because most of them are presenting with acute HF and rapidly deteriorate, so we decided not to include them. It is important to underline that our result should be considered only for children >1 year of age. Further studies are needed for this subgroup. Another point should be considered: in such a long follow-up, the adjustment of therapy over time was made, and in eight patients of the carvedilol group, ivabradine was added to carvedilol, as their LVEF did not increase above 40%. Although the improvement in ventricular function and the clinical outcome appears to be beneficial in those taking a higher dosage of carvedilol, larger studies are needed. The effect of carvedilol on HR should be considered in future studies for pediatric HF not only under a safety profile but also as an efficacy outcome. In our study, we obtained a reduction of HR of 30% from baseline, but more studies on the prognostic impact of HR in the pediatric population are warranted.

## Conclusions

Effective strategies for managing severe LV dysfunction in children with DCM that could reduce or delay the need for transplantation remain the goal of pharmacological therapy. The results of this study suggest that a high dosage of carvedilol regimen, in accordance with previous observations of PK in children, could improve clinical outcomes. The sustained beneficial effect of carvedilol remains over the years.

## Data Availability Statement

The raw data supporting the conclusions of this article will be made available by the authors, without undue reservation.

## Author Contributions

RA and NC contributed to conception and design of the study. NC organized the database. NC and MC performed the statistical analysis. RA wrote the first draft of the manuscript. RA, NC, and MC wrote sections of the manuscript. All authors contributed to manuscript revision, read, and approved the submitted version.

## Conflict of Interest

The authors declare that the research was conducted in the absence of any commercial or financial relationships that could be construed as a potential conflict of interest.

## Publisher's Note

All claims expressed in this article are solely those of the authors and do not necessarily represent those of their affiliated organizations, or those of the publisher, the editors and the reviewers. Any product that may be evaluated in this article, or claim that may be made by its manufacturer, is not guaranteed or endorsed by the publisher.
